# Integrating Optical Imaging Tools for Rapid and Non-invasive Characterization of Seed Quality: Tomato (*Solanum lycopersicum* L.) and Carrot (*Daucus carota* L.) as Study Cases

**DOI:** 10.3389/fpls.2020.577851

**Published:** 2020-12-21

**Authors:** Patrícia A. Galletti, Marcia E. A. Carvalho, Welinton Y. Hirai, Vivian A. Brancaglioni, Valter Arthur, Clíssia Barboza da Silva

**Affiliations:** ^1^Department of Crop Science, College of Agriculture “Luiz de Queiroz”, University of São Paulo, Piracicaba, Brazil; ^2^Department of Genetics, College of Agriculture “Luiz de Queiroz”, University of São Paulo, Piracicaba, Brazil; ^3^Department of Exacts Sciences, College of Agriculture “Luiz de Queiroz”, University of São Paulo, Piracicaba, Brazil; ^4^Laboratory of Radiobiology and Environment, Center for Nuclear Energy in Agriculture, University of São Paulo, Piracicaba, Brazil

**Keywords:** chlorophyll fluorescence, multispectral imaging, random forest, chemometrics, machine learning, seedlots, seed physiological potential, photosynthesis

## Abstract

Light-based methods are being further developed to meet the growing demands for food in the agricultural industry. Optical imaging is a rapid, non-destructive, and accurate technology that can produce consistent measurements of product quality compared to conventional techniques. In this research, a novel approach for seed quality prediction is presented. In the proposed approach two advanced optical imaging techniques based on chlorophyll fluorescence and chemometric-based multispectral imaging were employed. The chemometrics encompassed principal component analysis (PCA) and quadratic discrimination analysis (QDA). Among plants that are relevant as both crops and scientific models, tomato, and carrot were selected for the experiment. We compared the optical imaging techniques to the traditional analytical methods used for quality characterization of commercial seedlots. Results showed that chlorophyll fluorescence-based technology is feasible to discriminate cultivars and to identify seedlots with lower physiological potential. The exploratory analysis of multispectral imaging data using a non-supervised approach (two-component PCA) allowed the characterization of differences between carrot cultivars, but not for tomato cultivars. A Random Forest (RF) classifier based on Gini importance was applied to multispectral data and it revealed the most meaningful bandwidths from 19 wavelengths for seed quality characterization. In order to validate the RF model, we selected the five most important wavelengths to be applied in a QDA-based model, and the model reached high accuracy to classify lots with high-and low-vigor seeds, with a correct classification from 86 to 95% in tomato and from 88 to 97% in carrot for validation set. Further analysis showed that low quality seeds resulted in seedlings with altered photosynthetic capacity and chlorophyll content. In conclusion, both chlorophyll fluorescence and chemometrics-based multispectral imaging can be applied as reliable proxies of the physiological potential in tomato and carrot seeds. From the practical point of view, such techniques/methodologies can be potentially used for screening low quality seeds in food and agricultural industries.

## Introduction

Food quality and safety are the most important aspects in food and agricultural industries, which have been revolutionized by the development of more sophisticated, accurate and rapid testing methods that are mainly based on advanced optical imaging. Biological imaging of unaltered samples can be performed with non-destructive and real-time visualization of physical, chemical, physiological and pathological attributes of products, revealing internal quality of agricultural commodities and various food products, including grain, fruits, vegetables, meat, and fish (Wu and Sun, 2013; Rahman and Cho, 2016; Kumar and Karne, 2017). Methods based on electromagnetic properties of seed tissues, such as chlorophyll fluorescence and multispectral imaging fit well into these desirable features. Chlorophyll fluorescence-based technology is centered on the capacity of chlorophyll, which is often present in seeds during their development to emit light in a slightly longer wavelength in relation to the light that was absorbed (Misra et al., 2012; Smolikova et al., 2017). Because chlorophyll degradation is usually observed at late steps of seed maturation (Smolikova et al., 2020), chlorophyll content and its fluorescence are inversely proportional to the seed maturity and, consequently, to its quality (Ooms and Destain, 2011).

Multispectral imaging is a non-destructive technology able to integrate the conventional vision and spectroscopy technique to obtain at same time spatial and spectral information of an object (Shrestha et al., 2015; Mastrangelo et al., 2019; França-Silva et al., 2020), with accurate measurements of uniform and non-homogeneous samples. Advanced multispectral imaging systems have the great advantage to assess simultaneously multiple components, providing information about texture, color, shape, size and chemical composition for quality assurance. The basic principle of this technique is that all types of materials reflect and absorb electromagnetic energy in different patterns at specific wavelengths because of the difference in their physical structure and chemical composition. However, considering that multispectral images are a collection of images at various bands of spectrum, they provide massive information in the spatial and spectral domains. Thus, there are still some challenges regarding data interpretation and analysis; in such situations, mathematical chemometric models can underpin the dominant patterns in large data matrices in a fast and robust manner (ElMasry et al., 2019).

With the latest optical imaging features, we decided to develop a light-based method for characterization of seed quality by means of its physiological potential. It is well-known that crop success depends primarily on high-quality seeds (i.e., with high physiological potential), which have an increased germinability and elevated potential for generation of vigorous seedlings. An increased and fast germination provides high number of seedlings that cover the soil rapidly, not only promoting the formation of homogeneous stand but also increasing the seedling potential for using more efficiently radiation and nutrients in relation to weeds (Atkinson et al., 2019). Additionally, vigorous seedlings present an improved capacity to endure stressing conditions (Hemender et al., 2018). Nevertheless, seed quality assessment is currently centered on germination and vigor tests that are destructive, time consuming, labor intensive, and requiring experienced seed analysts. Therefore, we investigated the capacity of chlorophyll fluorescence and multispectral reflectance-based technologies for quality characterization of commercial seedlots. Among plants that are relevant as both crops and scientific models, tomato (*Solanum lycopersicum* L.) and carrot (*Daucus carota* L.) were selected for the experiment.

## Materials and Methods

### Plant Materials

Different commercial lots of tomato and carrot seeds were investigated (Table 1). All seedlots were kept at 10°C during the experimental period. Seed moisture content (fresh weight basis) was 6.4–6.9 and 5.3–5.6% in seedlots of “Gaúcho” and “Tyna” tomato, respectively, and 6.5–8.2 and 7.1–7.7 in seedlots of “Brasília” and “Francine” carrot, respectively.

**Table 1:** Details of tomato and carrot seeds used in this study.

**Tomato**	**Seed features**					**Storage**	
	**Cultivar**	**Lot**	**Harvest year**	**Seed source**	**Calibration**	**Temperature (°C)**	**Period (months)**
	Gaúcho (G)	G-I	2016	BP, Brazil	Non-calibrated	10	24
		G-II	2015	Yuba, USA	Non-calibrated	18	36
		G-III	2015	Yuba, USA	Non-calibrated	18	36
	Tyna (T)	T-IV	2015	BP, Brazil	3.1^a^	17	26
		T-V	2014	BP, Brazil	3.2	17	36
		T-VI	2014	BP, Brazil	2.6	17	36
		T-VII	2015	BP, Brazil	2.5	17	26
**Carrot**						
	Brasília (B)	B-I	2017	Candiota, Brazil	1.6–2.2^b^	18	12
		B-II	2017	Candiota, Brazil	1.6–2.2	18	12
		B-III	2017	Candiota, Brazil	1.6–2.2	18	12
		B-IV	2017	Candiota, Brazil	1.6–2.2	18	12
	Francine (F)	F-V	2015	Longiano, Italy	2.4–2.6	14	36
		F-VI	2015	Longiano, Italy	1.4–1.6	14	36
		F-VII	2015	SVT, Chile	2.2–2.4	14	36
		F-VIII	2015	SVT, Chile	1.4–1.6	14	36

*BP, Bragança Paulista; SVT, San Vicente de Tagua Tagua*.

a *Weight (g) of 1,000 seeds*.

b *Seed size (mm)*.

### Traditional Tests to Rank Lots According to Seed Physiological Potential

Germination and vigor tests were performed to rank quality of lots based on seed performance. The germination tests were evaluated at 7 and 14 days with four replications of 50 seeds per lot. Seeds were sown on blotting paper moistened with distilled water (1: 2.5, g: mL) placed inside transparent plastic box (11.0 × 11.0 × 3.5 cm) and kept at daily temperature alternations (8 h at 30°C with light and 16 h at 20°C in the dark). Seed vigor was estimated based on (i) germination at 7 days (early germination test), (ii) germination after accelerated aging, (iii) seedling emergence at room temperature, (iv) emergence speed index, (v) vigor index, and (vi) seedling length.

The accelerated aging test was conducted with 200 seeds distributed over a single layer on a wire mesh screen suspended inside a box (11.0 × 11.0 × 3.5 cm) containing 40 mL of saturated NaCl solution (40 g NaCl 100 ml^−1^ of water). Lid-covered boxes were maintained at 41°C for 72 h; next, four replications of 50 seeds were tested for germination, and data were recorded 7 days after sowing. Seedling emergence and emergence speed index were evaluated using four replications with 50 seeds. Seeds were sown in polystyrene trays containing a commercial substrate based on a mix of pine bark, peat moss and vermiculite. The trays were kept under room conditions for 14 days. The emergence speed index was based on the daily record of seedlings emerged from the substrate (Maguire, 1962).

The vigor indexes and seedling length were calculated by the Seed Vigor Imaging System™, SVIS software (Ohio State University, Columbus, OH). Four replications of 25 seeds per lot were distributed horizontally in two rows in the upper-third of the surface of moistened blotting paper placed within transparent plastic box (11.0 × 11.0 × 3.5). Tomato seeds were kept in a germination chamber at 25°C for 4 days, and carrot seeds were maintained at 20°C for 6 days; subsequently, seedlings were scanned and analyzed by the SVIS software. The vigor index was calculated by combining the growth parameters (70% of contribution) with seedling uniformity (30% of contribution), both based on the maximum seedling length at 4 and 6 days after sowing for tomato (9.0 cm) and carrot (5.1 cm), respectively, as established in preliminary tests.

### Chlorophyll Fluorescence Imaging

Chlorophyll fluorescence images of the seeds were captured and analyzed using two instruments: SeedReporter™ (PhenoVation B.V., Wageningen, Netherlands) and VideometerLab4™ (Videometer A/S, Herlev, Denmark). SeedReporter™ instrument provides excitation of the chlorophyll complex by high intensity amber colored light. Chlorophyll excitation is induced by illumination of the seeds at 620 nm and the chlorophyll fluorescence signals are detected at 730 nm. Three plates with 100 seeds per lot were positioned at 20.0 cm from 36 high intensity amber Light-Emitting Diode (LED). A charge-coupled device chip (CCD-chip) was used for fluorescence detection and the signal was transmitted to a 14-bit analog-to-digital converter. Before image acquisition, the light setup was adjusted to avoid overload. High-resolution chlorophyll fluorescence image (2448 × 2448 pixels) was acquired during 1 s integration time, requiring no sample preparation. Chlorophyll fluorescence images were analyzed by the SeedReporter™ software version 5.4.6. Seeds were masked from the background and identified as a region of interest, which allowed the calculation of chlorophyll fluorescence mean for individual seeds and per lot.

In the VideometerLab4™, chlorophyll excitation was induced by illumination of the seeds at 630, 645, and 660 nm, and an emission filter 700 LP measured the chlorophyll fluorescence. This instrument consists of a sphere with a matte white titanium coating to ensure that light is scattered evenly around the object, in which LEDs are placed at sphere rim, and a CCD sensor is mounted in the top of the sphere. The three plates with 100 seeds analyzed by SeedReporter™ were transferred to three 9-cm glass Petri dishes. The VideometerLab4™ captured high-resolution chlorophyll fluorescence images (2192 × 2192 pixels) in a few seconds, requiring no sample preparation. Chlorophyll fluorescence images were analyzed by the VideometerLab™ software version 3.14.9. A image segmentation technique based on thresholding was applied so that the seeds were completely separated from the background. Seeds were extracted into a Binary Large Object (BLOB) toolbox, a built-in function in VideometerLab software; each BLOB was a representation of one seed, and the chlorophyll fluorescence was measured for individual seeds and per lot.

### Multispectral Imaging

Multispectral images were captured at 19 wavelengths−365 (UVA), 405 (violet), 430 (indigo), 450 (blue), 470 (blue), 490 (cyan), 515 (green), 540 (green), 570 (yellow), 590 (amber), 630 (red), 645 (red), 660 (red), 690 (deep red), 780 (deep red), 850, 880, 940, and 970 nm (the last four wavelengths in the NIR region), using a VideometerLab4™ instrument (Videometer A/S, Herlev, Denmark) and its software (version 3.14.9). This system can capture multispectral images combining them into high-resolution multispectral images (2192 × 2192 pixels). Three replications of 100 seeds per lot were placed in 9-cm glass Petri dishes. Before image acquisition, the light setup was adjusted to optimize the light intensity in each bandwidth, resulting in an improved signal-to-noise ratio in such a way that the images captured could be directly comparable. Light setup was adjusted using a representative sample, then the strobe time of each illumination type was optimized with respect to this area. Subsequently, the system was calibrated using three calibration targets: (i) uniform bright disc, (ii) uniform dark disc, and (iii) geometric disc which is black with dots in a rectangular grid. The samples were placed under an integrating sphere with uniform diffuse lighting using 19 LEDs positioned side by side around the rim of the sphere. The curvature of the sphere and its matt-white coating ensure a uniform reflection of the cast light. After successive illumination of the sample with 19 LEDs (sequential strobes), multispectral images were captured in one sequence during 5 s, requiring no sample preparation. Each seed was identified as a region of interest to segment the seeds from the background, and seeds were extracted into a BLOB.

**Figure 1:**
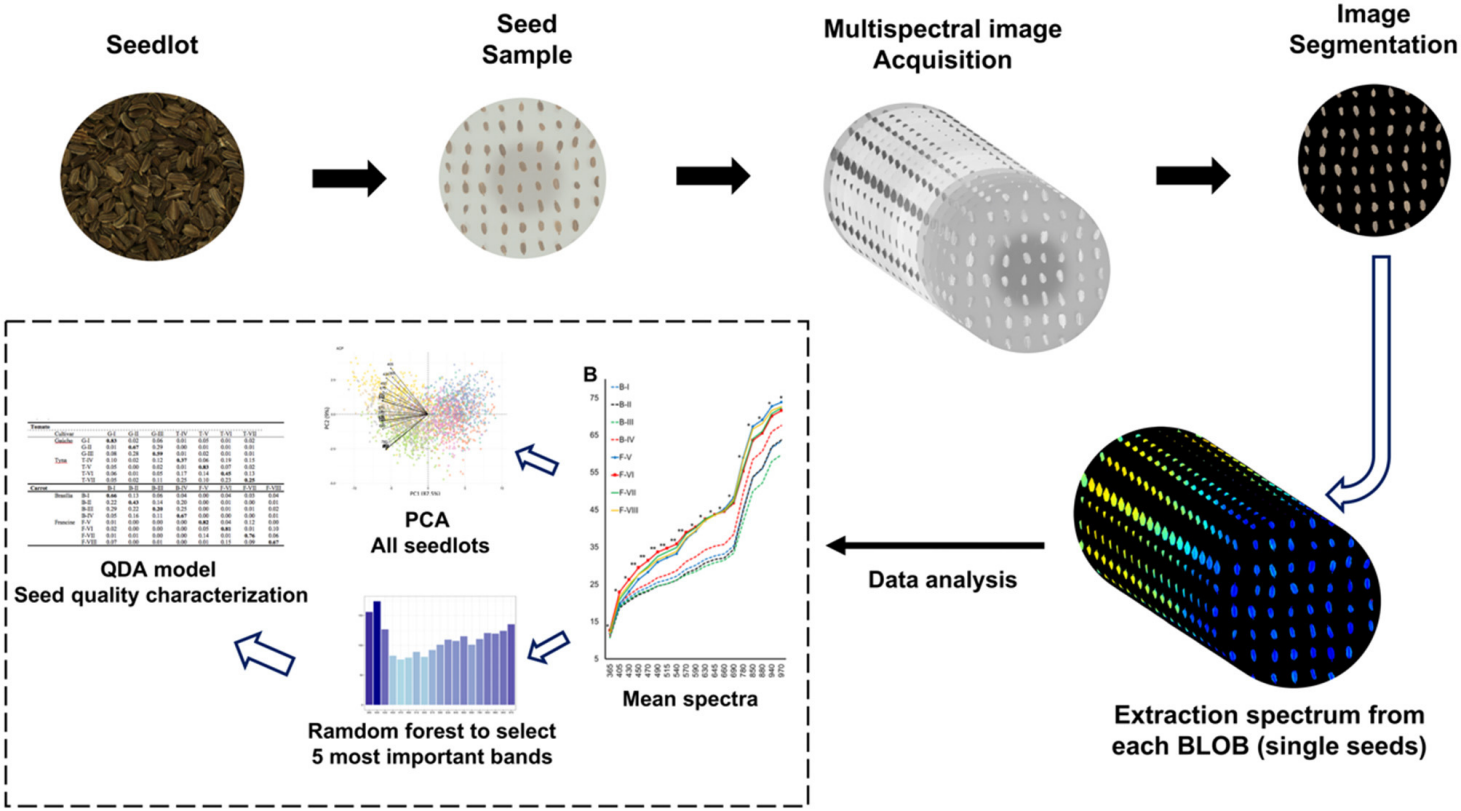
Flowchart of the main procedure for extraction and analyzing multispectral imaging data.

Mean spectra were plotted to show the difference among the seedlots based on their multispectral patterns. We applied two chemometric tools to process multispectral data, (i) principal component analysis (PCA) and (ii) quadratic discrimination analysis (QDA) (Figure 1). A biplot using the first two principal components (PC1 and PC2) was built to characterize the cultivars and seedlots based on mean reflectance at 19 wavelengths. A Random Forest (RF) model (Liaw and Wiener, 2002) was built with 1,000 trees to rank the importance of wavelengths to discriminate the seedlots. The classification of the most important wavelengths was based on Gini coefficient; the higher the value the more important the band in classification of the RF (Izenman, 2013). In order to validate the RF model, multispectral data corresponding to the five most important wavelengths, as previously assigned by the RF classifier were used in a QDA model. The QDA model (Venables and Ripley, 2002) was applied for discrimination of different seedlots and low-and high-vigor seedlots. The calibration set comprised 70% of the data, and the remaining 30% were used to validate the model. The statistical analyses were performed using R software version 3.6.2 (R Core Team, 2019). Data analysis were performed with packages ggplot2 (Wickham, 2016), reshape2 (Wickham, 2007) and factoextra (Kassambara and Mundt, 2019).

### X-Ray Imaging

Radiographic images of all samples were generated to identify immature seeds, and they were used for illustration only. X-ray images were acquired using the MultiFocus™ instrument (Faxitron Bioptics LLC, USA). This system is integrated with advanced automatic exposure control to automatically select the appropriate exposure time and kV settings for the sample. The specific time and voltage settings were saved for all subsequent images with 4.9 s and 25 kV in “Gaúcho” and “Tyna” tomato, 5.0 s and 25 kV in “Brasília” carrot, and 4.7 s and 26 kV in “Francine'' carrot.

### Photosynthesis and Chlorophyll Content in Seedlings

Chlorophyll fluorescence-based technology was applied to obtain information in seedlings using a SeedReporter™ (PhenoVation B.V., Wageningen, Netherlands). This system allows the imaging of photosynthetic parameters of whole plants within a short time interval (≈800 ms). Seeds were germinated in polystyrene pots 500 mL of volume (eight pots per treatment), filled with a mix of pine bark, peat moss and vermiculite; each compartment hosted 10 seeds. Seedlings were grown in a controlled chamber with air temperature of 25°C and 50–70% of relative humidity and artificial light only (LED lamps, 13 W), with a photoperiod of 8/16 h of light/dark. At the beginning of the experiment, the photosynthesis active radiation (PAR) was measured using a Quantum PAR meter (Spectrum Tecnologies, 3415FSE, Illinois, United States), which was 150 μmols m^−2^ s^−1^ from a distance of 13.0 cm from the pots. When the seedlings were well-established, 7 days after sowing, they were thinned to eliminate overlapping.

Measurements were conducted in 32 seedlings from low-and high-vigor seedlots (eight pots with four seedlings per treatment) taken at 14 and 21 days after sowing. A saturation of the photosynthesis process was provided by 36 high intensity amber LED sources (peak at 620 nm), imposing an intensive light flash of 6,320 μmol m^−2^ s^−1^ using the Kautsky induction curve technology (Strasser et al., 1995). The leaves were positioned at 25 cm from the LEDs. An interference filter (730 nm) blocks the LED sources and transmits the chlorophyll fluorescence from the plant tissue onto the CCD-chip. High-resolution images (2448 × 2448 pixels) of dark-adapted seedlings were acquired for photosynthetic activity by calculation of the variable fluorescence over saturation level of fluorescence (F_V_/F_M_). Using the SeedReporter™ software version 5.4.6., each pixel of the fluorescence image F_V_/F_M_ was calculated, which correlates with the quantum yield of Photosystem II (PSII) photochemistry. A broad-band white (3,000 K) in a range of ~450–780 nm was used to calculate the reflectance mean of leaves, measured with an optical filter of NIR (770 nm). The software estimated the chlorophyll index from reflectance at 710 and 770 nm: chlorophyll index = (reflectance at 770 nm/reflectance at 710 nm)-1 (Gitelson et al., 2003).

## Results

### Seed Physiological Potential by Traditional Analytical Methods

**Figure 2:**
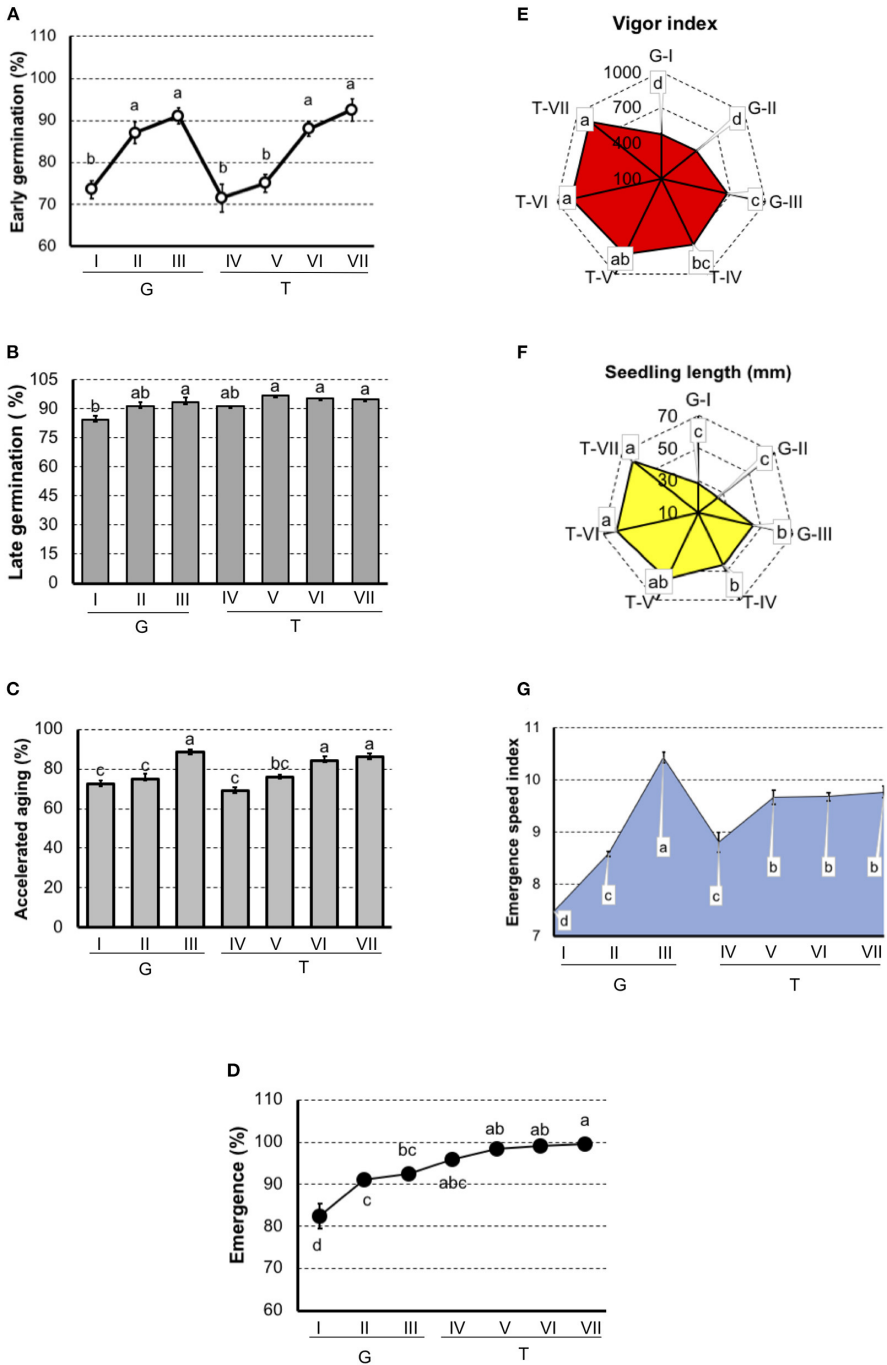
Germination and vigor tests to rank tomato seedlots of Gaúcho (G-I, G-II, and G-III) and Tyna (T-IV, T-V, T-VI, and T-VII) cultivar. **(A)** Germination at 7 days after sowing. **(B)** Germination at 14 days after sowing. **(C)** Germination of artificially aged seeds. **(D)** Seedling emergence at room temperature 14 days after sowing. **(E)** Vigor index based on the speed and uniformity of tomato seedling in relation to the estimated maximum 4-d-old-seedlings. **(F)** Seedling length 4-d old. **(G)** Emergence speed index. Means (± SE) with a common letter are not significantly different (*P* > 0.05).

In tomato (Figure 2), results showed that G-I had the lowest physiological potential, particularly in the seedling emergence test and the emergence speed index. In “Gaúcho” tomato, the G-III lot presented the highest vigor when evaluated by the accelerated aging test, vigor index, seedling length, and emergence speed index. In “Tyna” tomato, T-IV and T-V lots were separated as lower performance in the early germination and accelerated aging tests, and these methods also separated T-VI and T-VII with the best physiological potential.

**Figure 3:**
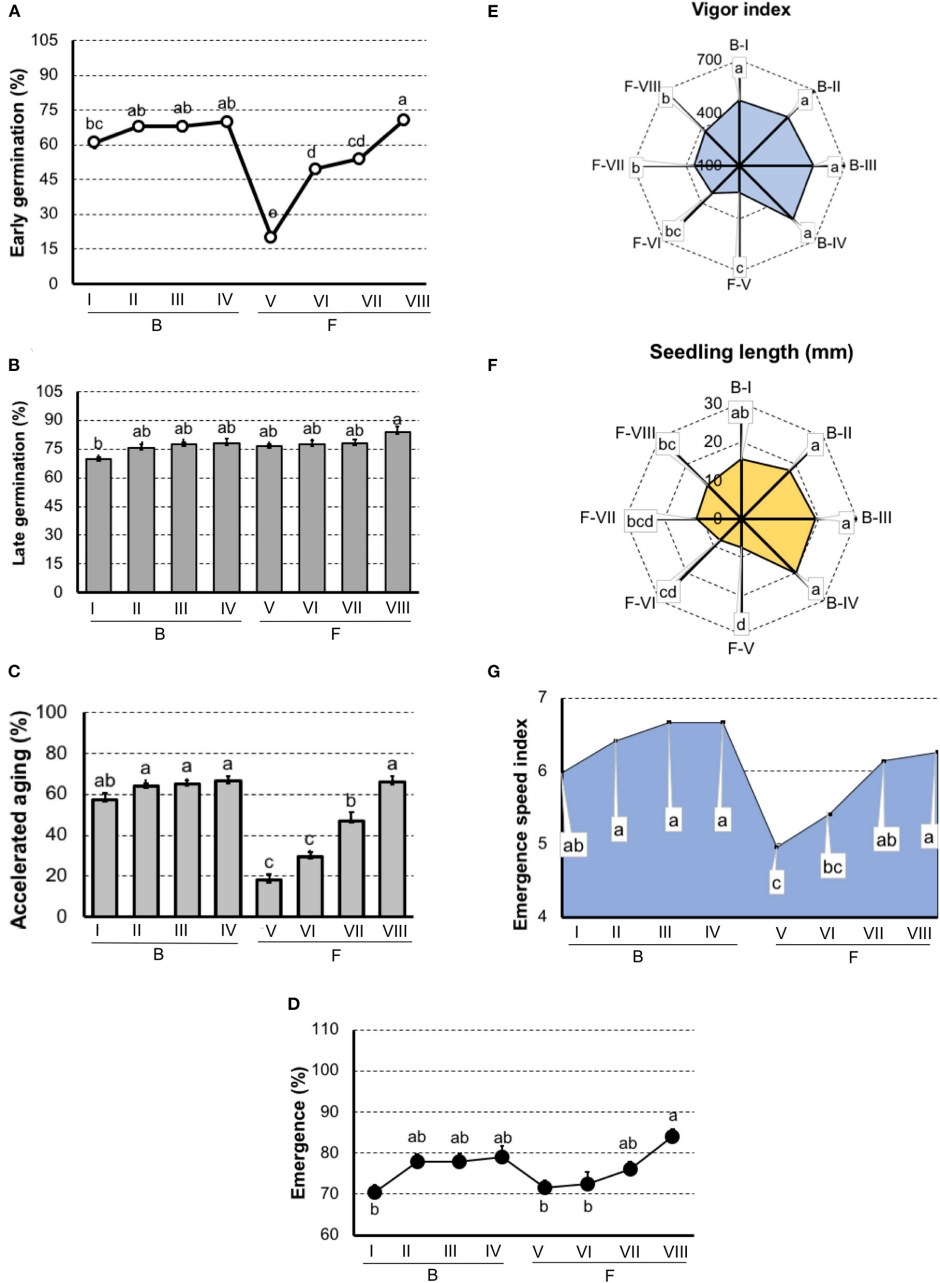
Germination and vigor tests to rank carrot seedlots of Brasília (B-I, B-II, B-II, and B-IV) and Francine (F-V, F-VI, F-VII, and F-VIII) cultivar. **(A)** Germination at 7 days after sowing. **(B)** Germination at 14 days after sowing. **(C)** Germination of artificially aged seeds. **(D)** Seedling emergence at room temperature 14 days after sowing. **(E)** Vigor index based on the speed and uniformity of carrot seedling in relation to the estimated maximum 6-d-old-seedlings. **(F)** Seedling length 6-d old. **(G)** Emergence speed index. Means (± SE) with a common letter are not significantly different (*P* > 0.05).

In carrot (Figure 3), both germination and vigor tests did not detected differences among lots in “Brasília.” But, in “Francine,” the early germination test showed sensibility to classify seeds with the lowest (F-V) and greatest vigor (F-VIII).

### Chlorophyll Fluorescence in Seeds

**Figure 4:**
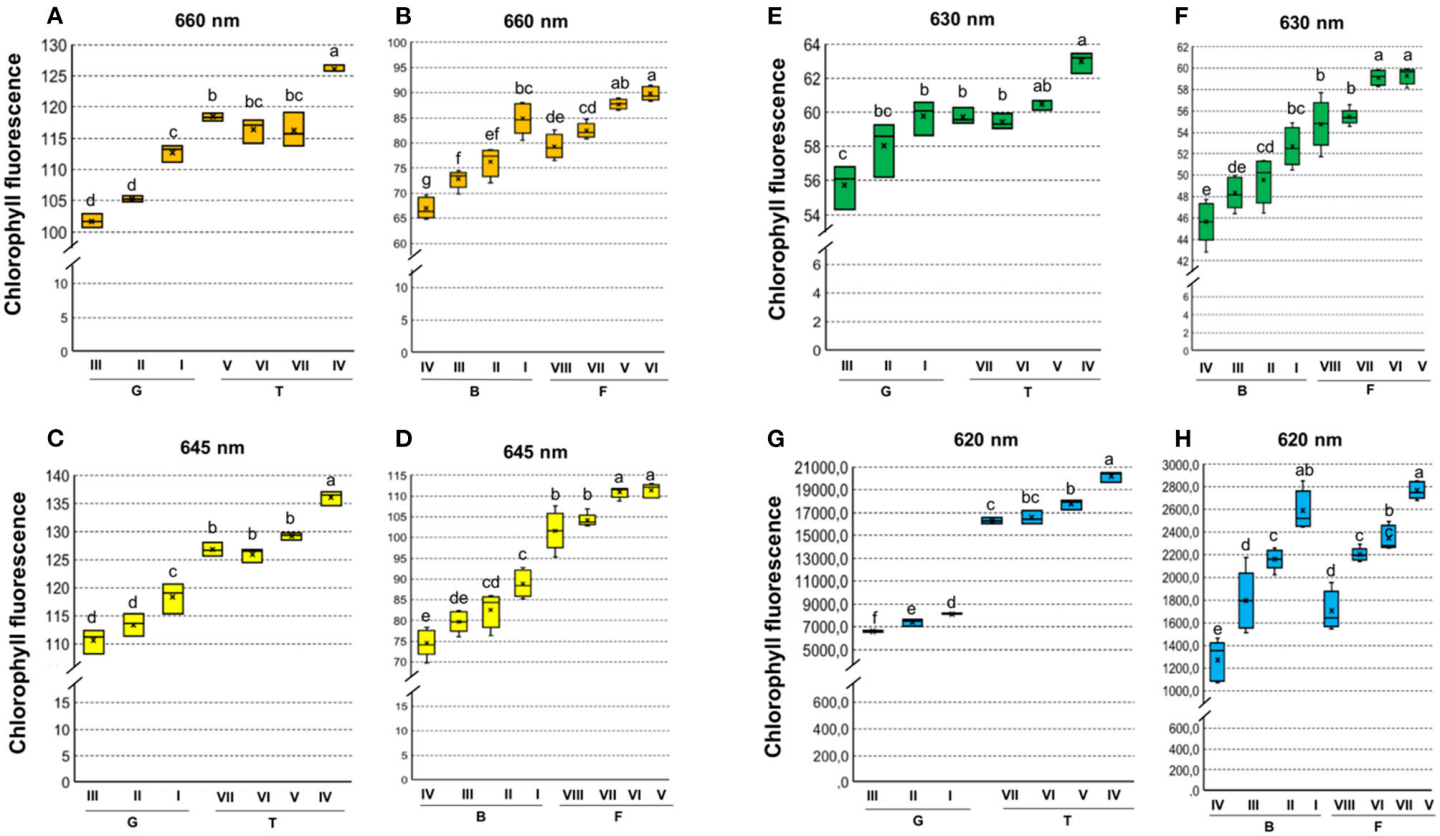
Histogram of chlorophyll fluorescence in seedlots of tomato **(A,C,E,G)** and carrot **(B,D,F,H)** at 660/700, 630/700, 645/700,and 620/730 nm excitation-emission. Tomato lots are represented by Gaúcho (G) and Tyna (T) cultivars: G-I, G-II, G-III, T-IV, T-V, T-VI. Carrot lots are represented by Brasília (B) and Francine (F) cultivars: B-I, B-II, B-II, B-IV, F-V, F-VI, F-VII, and F-VIII. Means with a common letter are not significantly different (*P* > 0.05) (*n* = 300).

**Figure 5:**
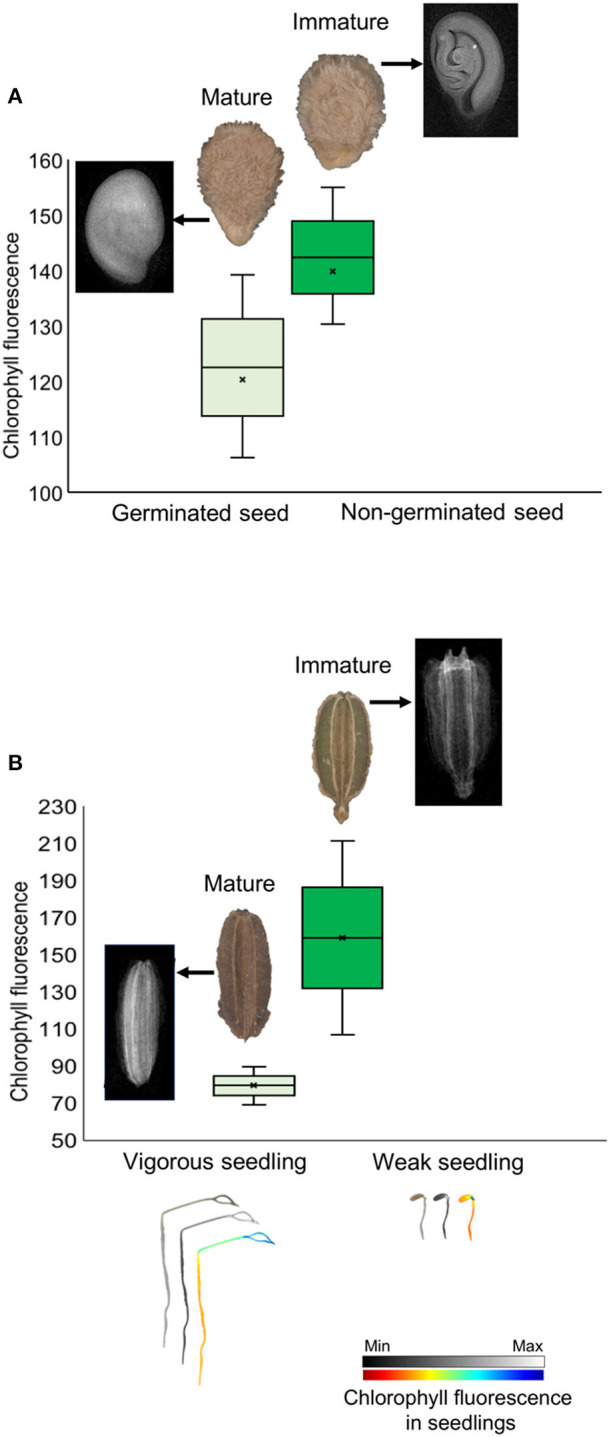
Histogram of chlorophyll fluorescence in mature and immature seeds of tomato **(A)** and carrot **(B)** at 660/700 nm (excitation-emission). Radiographic images show empty spaces (low amount of stored reserves) in seeds with high chlorophyll fluorescence because of their immaturity, with formation of non-viable seeds in tomato and weak seedlings in carrot (with low chlorophyll fluorescence signal in seedlings).

Chlorophyll fluorescence analysis at 620/730 nm excitation-emission allows to separate tomato cultivars (Figure 4G), and particularly at 645/700 nm discriminated cultivars in both tomato and carrot (Figures 4C,D). High fluorescence values coincided with low-quality seed lots (Figure 4), which were better discriminated when 620/730 nm (Figure 4G) and 660/700 nm (Figure 4B) were used for chlorophyll excitation in tomato and carrot seeds, respectively. Radiographic imaging revealed that seeds with high chlorophyll fluorescence presented empty spaces (i.e., lower amounts of stored reserves) (Figure 5), and these seeds were non-viable or generated weak seedlings with low chlorophyll fluorescence signal in their tissues.

### Multispectral Imaging-Based Chemometric Analysis

**Figure 6:**
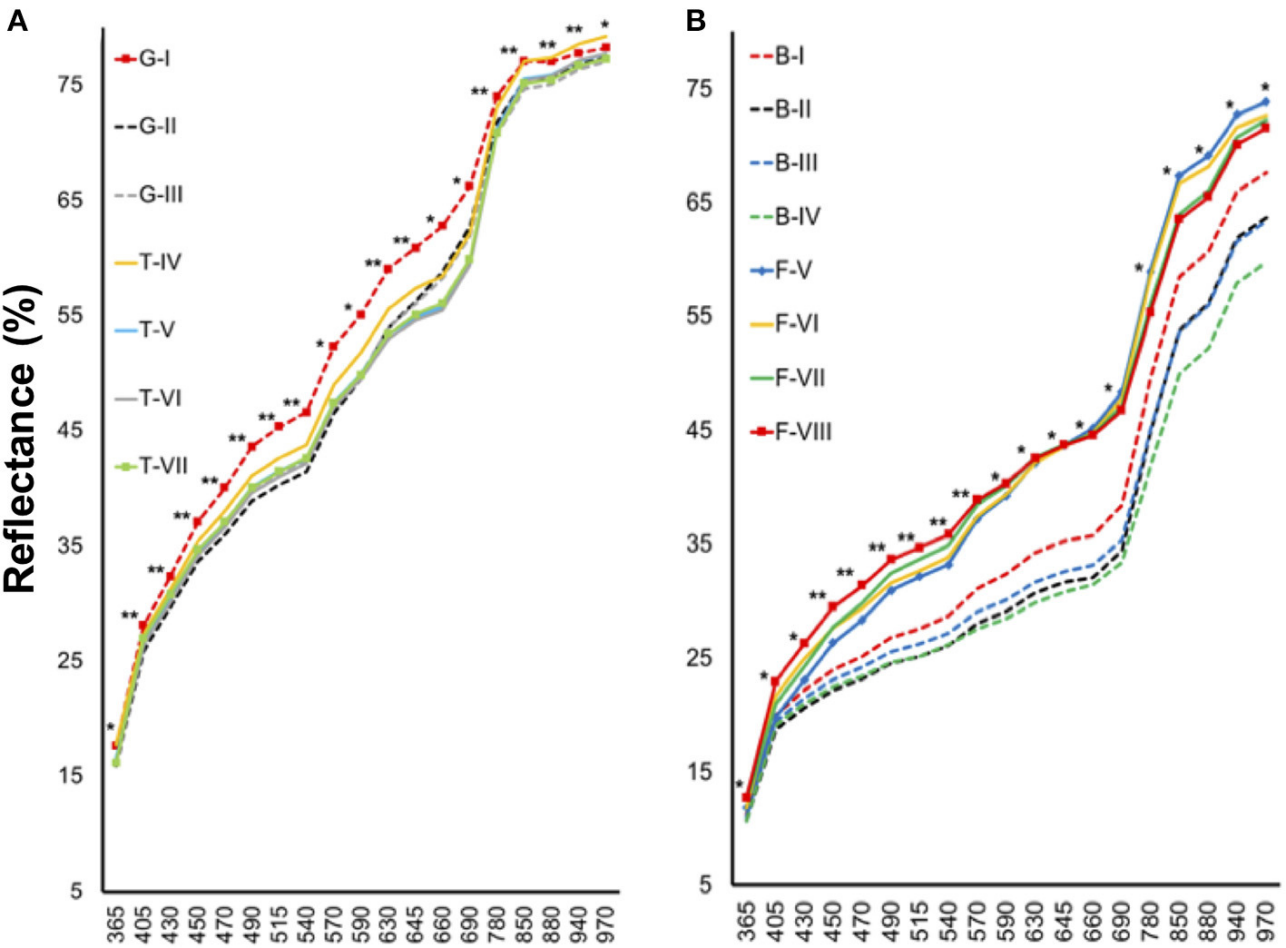
Spectral signature of tomato **(A)** and carrot **(B)** seedlots at 19 wavelengths in a range from 365 to 970 nm. Tomato lots are represented by Gaúcho (G) and Tyna (T) cultivars: G-I, G-II, G-III, T-IV, T-V, T-VI. Carrot lots are represented by Brasília (B) and Francine (F) cutivars: B-I, B-II, B-II, B-IV, F-V, F-VI, F-VII, and F-VIII; *, **significant at the 0.05 and 0.01 probability levels (*n* = 300).

**Figure 7:**
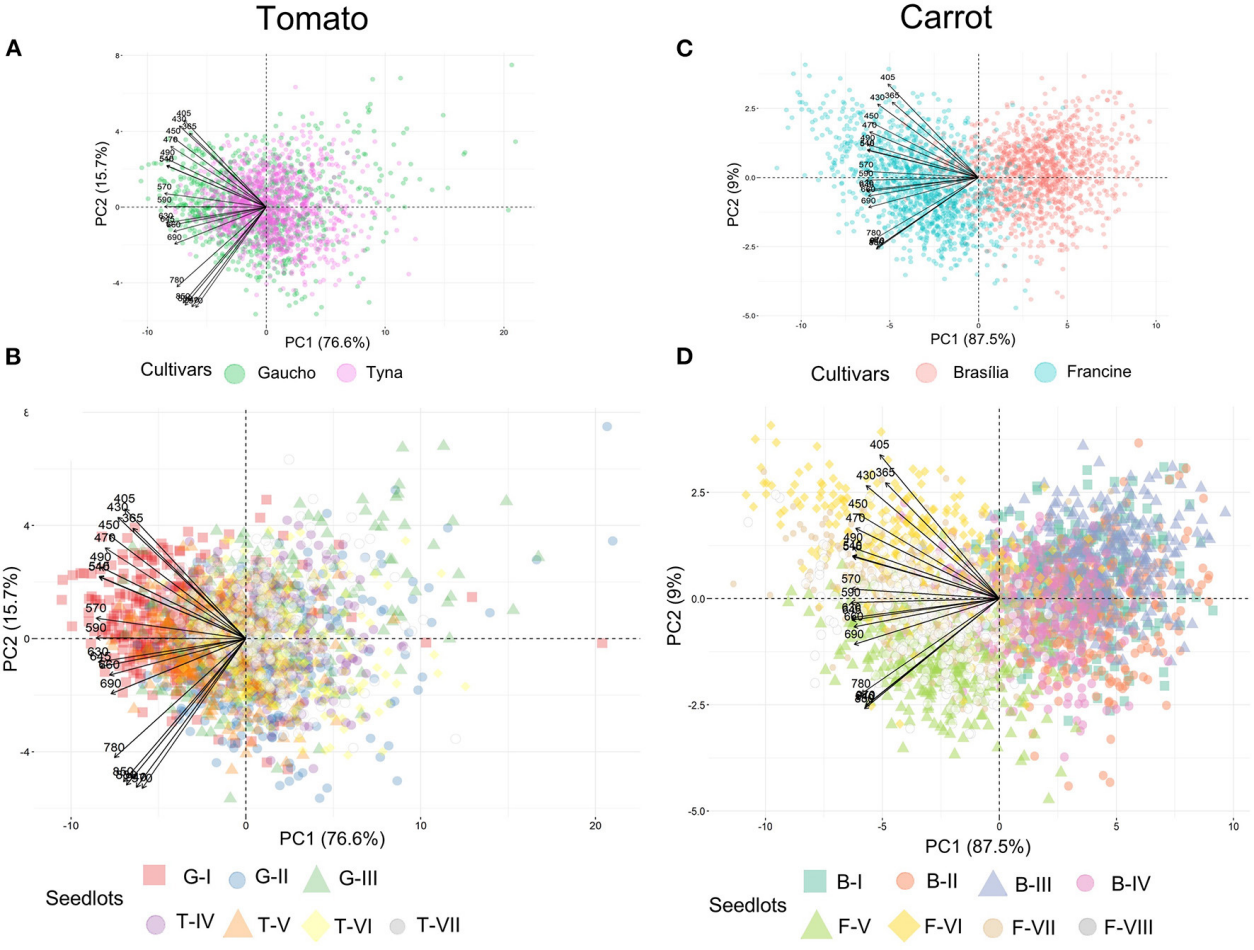
Biplots of principal component analyses for multispectral reflectance in tomato **(A,B)** and carrot **(C,D)** seedlots at 19 wavelengths in a range from 365 to 970 nm. Tomato lots are represented by Gaúcho (G) and Tyna (T) cultivars: G-I, G-II, G-III, T-IV, T-V, T-VI. Carrot lots are represented by Brasília (B) and Francine (F) seedlots: B-I, B-II, B-II, B-IV, F-V, F-VI, F-VII, and F-VIII (*n* = 300).

**Figure 8:**
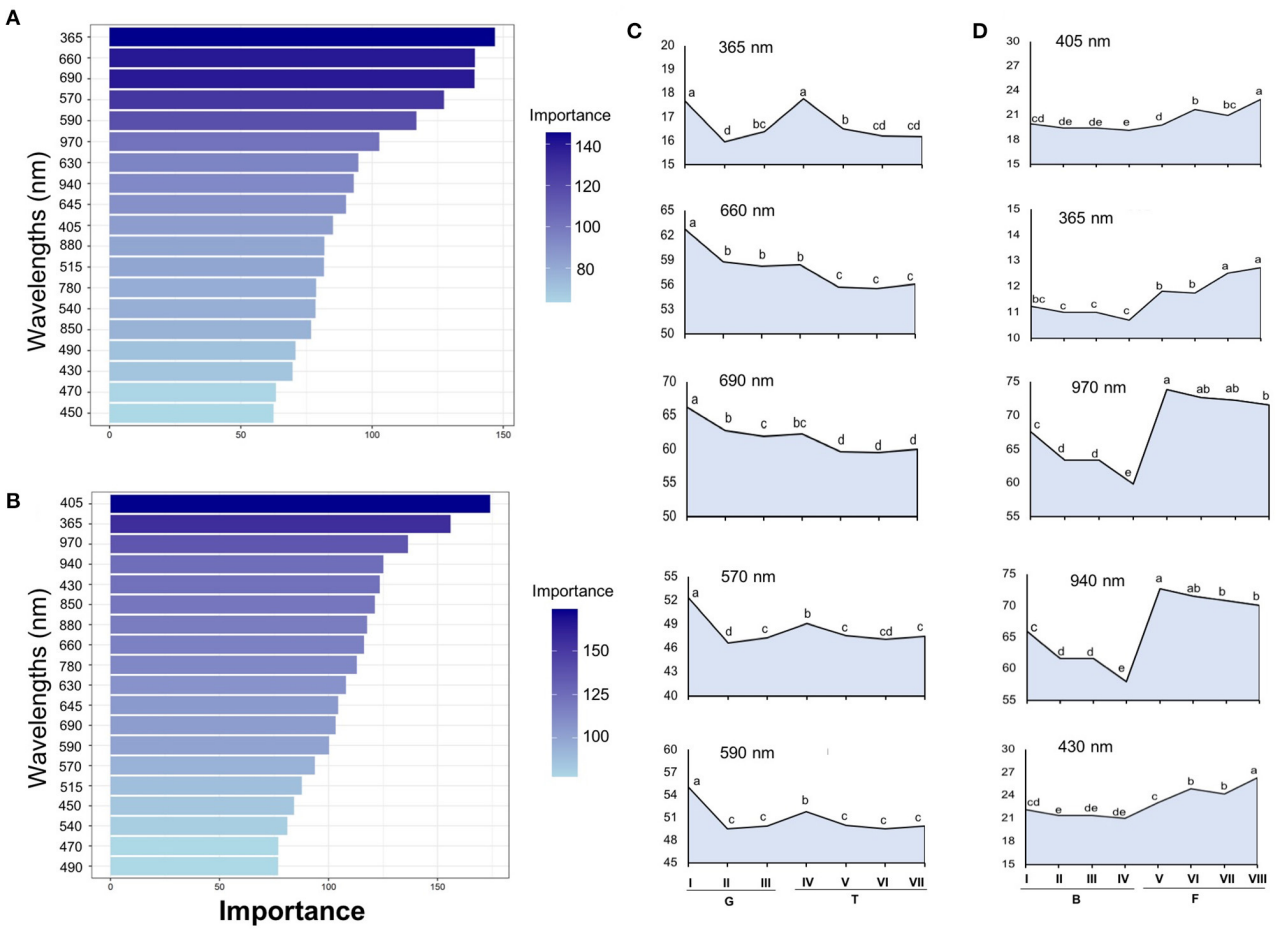
Gini-based importance for each wavelength in the Random Forest to discriminate seedlots of tomato **(A)** and carrot **(B)**. Multispectral reflectance data were acquired at 19 wavelengths in a range from 365 to 970 nm. Reflectance mean for the five most important wavelengths assigned by the RF classifier in tomato **(C)** and carrot **(D)**; means with a common letter are not significantly different (*P* > 0.05). Tomato lots are represented by Gaúcho (G) and Tyna (T) cultivars: G-I, G-II, G-III, T-IV, T-V, T-VI. Carrot lots are represented by Brasília (B) and Francine (F) seedlots: B-I, B-II, B-II, B-IV, F-V, F-VI, F-VII, and F-VIII (*n* = 300).

**Figure 9:**
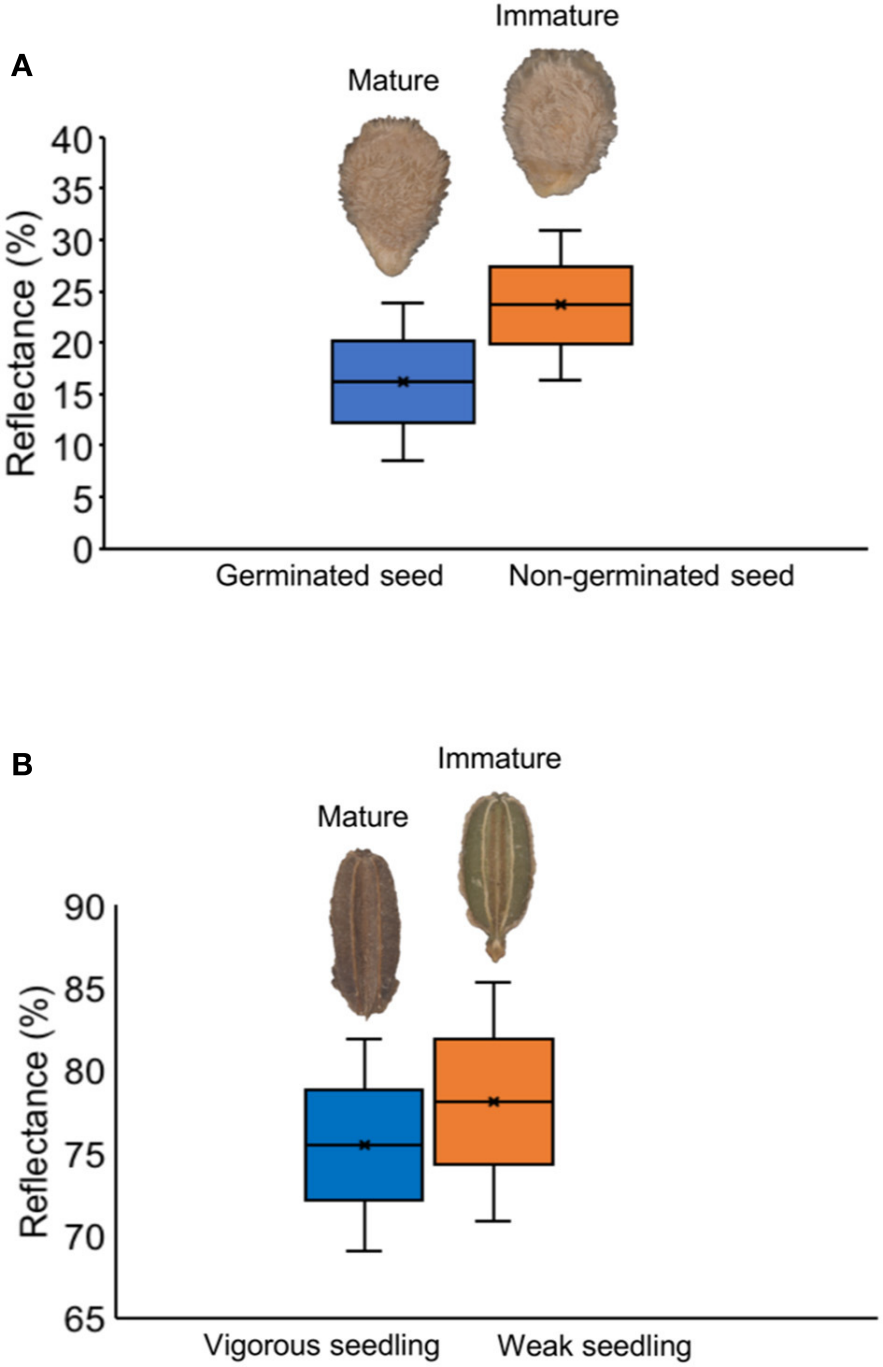
Histogram of reflectance in mature and immature seeds of tomato at 365 nm **(A)**, and carrot at 970 nm **(B)**.

Tomato lots with low-quality seeds (G-I and T-IV) presented the highest spectral signature (Figure 6A). In carrot, this pattern was clearly noticed between cultivars: “Francine” lots showed higher reflectance mean than “Brasília” (Figure 6B). Moreover, although the conventional testing techniques did not indicate differences among “Brasília” lots (Figure 3), B-I had higher spectral signature than other lots of this cultivar (Figure 6B). In “Francine,” the lot identified with the lowest performance (F-V) (Figure 3) exhibited higher reflectance only at longer wavelengths, especially in the NIR region (Figure 6B). A PCA explained 92.3 and 96.5% of the spectral variation among tomato and carrot seedlots, respectively (Figure 7). PC1 represented at least 76.6% of the total variance among tomato seedlots (Figures 7A,B) in which the G-I group showed more negative values than others (Figure 7B). This group was better characterized using multispectral data from 570 to 690 nm. Cultivars of carrot were characterized in the PC1-PC2 space, where PC1 accounted for 87.5% of the total variation among the lots (Figures 7C,D); “Francine” seedlots exhibited more negative values than “Brasília” with a strong influence of intermediate wavelengths (570–690 nm). Meanwhile, the PCA method was not able to characterize tomato cultivars (Figures 7A,B). A RF algorithm was applied to the multispectral data to select the five most meaningful wavelengths based on Gini importance, which were 365, 660, 690, 570 and 590 nm in tomato seeds (Figure 8A), and 405, 365, 970, 940, and 430 nm in carrot seeds (Figure 8B). Higher reflectance values, in particular at 365 nm in tomato (Figure 8C), and at 940 and 970 nm in carrot (Figure 8D) coincided with low-vigor seedlots of tomato (G-I and T-IV) and carrot (F-V), respectively. These lots had more immature seeds, with high reflectance intensity at those wavelengths (Figure 9).

To validate the RF model, the reflectance data obtained from the five most meaningful wavelengths assigned by the RF were used in two QDA-based models for characterization of seed quality. The first model was developed using data from all seedlots (Table 2). In “Gaúcho” and “Tyna” tomato, this model provided high performance to predict lower-vigor seedlots (G-I and T-IV), with accuracies of 82 and 83% for validation set. In “Francine” carrot, the model showed higher accuracy to classify seeds of F-V (82%), F-VI (80%), and F-VII (81%). But, the model was unable to accurately predict vigor levels in “Brasília” lots (19–64%). Nevertheless, the classifier had considerably high precision to correctly distinguish tomato and carrot cultivars, with low rate of false-positive and false-negative between cultivars. The second QDA model was created for class membership of higher-and lower-vigor seeds (Table 3), and it achieved an accuracy of 86–95% in tomato, and 88–97% in carrot.

**Table 2:** Quadratic discriminant analysis based on multispectral reflectance at 365, 570, 590, 660, and 690 nm from different tomato seedlots, and at 365, 405, 430, 940, and 970 nm from different carrot seedlots (*n* = 300).

**Tomato**			**Calibration**							
	**Cultivar**		**G-I**	**G-II**	**G-III**	**T-IV**	**T-V**	**T-VI**	**T-VII**	
	Gaúcho	G-I	**0.83**	0.01	0.09	0.04	0.00	0.01	0.01	
		G-II	0.01	**0.66**	0.30	0.01	0.01	0.00	0.01	
		G-III	0.07	0.28	**0.60**	0.01	0.02	0.01	0.01	
	Tyna	T-IV	0.04	0.00	0.02	**0.86**	0.00	0.07	0.00	
		T-V	0.09	0.01	0.12	0.06	**0.34**	0.26	0.12	
		T-VI	0.07	0.01	0.05	0.14	0.13	**0.50**	0.10	
		T-VII	0.08	0.02	0.12	0.09	0.21	0.28	**0.20**	
			**Validation**							
	Gaúcho	G-I	**0.82**	0.03	0.06	0.08	0.01	0.00	0.00	
		G-II	0.00	**0.68**	0.28	0.01	0.01	0.01	0.00	
		G-III	0.06	0.32	**0.53**	0.06	0.00	0.01	0.01	
	Tyna	T-IV	0.05	0.01	0.00	**0.83**	0.04	0.06	0.00	
		T-V	0.15	0.00	0.17	0.06	**0.28**	0.25	0.09	
		T-VI	0.05	0.01	0.04	0.21	0.09	**0.44**	0.16	
		T-VII	0.04	0.02	0.06	0.11	0.29	0.24	**0.23**	
**Carrot**		**Calibration**								
	**Cultivar**		**B-I**	**B-II**	**B-III**	**B-IV**	**F-V**	**F-VI**	**F-VII**	**F-VIII**
	Brasília	B-I	**0.67**	0.11	0.06	0.04	0.00	0.04	0.04	0.04
		B-II	0.25	**0.37**	0.15	0.21	0.00	0.01	0.00	0.01
		B-III	0.30	0.18	**0.22**	0.25	0.00	0.00	0.01	0.03
		B-IV	0.04	0.15	0.11	**0.69**	0.00	0.00	0.00	0.01
	Francine	F-V	0.00	0.00	0.00	0.00	**0.82**	0.04	0.13	0.00
		F-VI	0.03	0.00	0.00	0.00	0.06	**0.80**	0.01	0.11
		F-VII	0.01	0.01	0.00	0.00	0.16	0.01	**0.75**	0.05
		F-VIII	0.07	0.00	0.01	0.00	0.01	0.15	0.07	**0.68**
			**Validation**							
	Brasília	B-I	**0.64**	0.19	0.02	0.04	0.00	0.02	0.02	0.06
		B-II	0.30	**0.37**	0.08	0.22	0.00	0.01	0.01	0.00
		B-III	0.29	0.24	**0.19**	0.25	0.00	0.01	0.01	0.01
		B-IV	0.06	0.16	0.13	**0.64**	0.01	0.00	0.00	0.00
	Francine	F-V	0.01	0.00	0.00	0.00	**0.82**	0.06	0.10	0.01
		F-VI	0.03	0.00	0.01	0.00	0.03	**0.80**	0.04	0.10
		F-VII	0.01	0.01	0.01	0.00	0.09	0.01	**0.81**	0.05
		F-VIII	0.07	0.00	0.00	0.00	0.01	0.11	0.14	**0.66**

*Values in bold indicate the hit rate in the quadratic discriminant analysis*.

**Table 3:** Quadratic discriminant analysis based on multispectral reflectance at 365, 570, 590, 660, and 690 nm from tomato seedlots, and at 365, 405, 430, 940, and 970 nm from carrot seedlots for classes of lower and higher vigor seeds (*n* = 300).

**Tomato cultivars**		**Calibration**						
		**Gaúcho**					**Tyna**	
			**G-I**	**G-III**			**T-IV**	**T-VI**
		G-I	**0.93**	0.07		T-IV	**0.92**	0.08
		G-III	0.11	**0.89**		T-VI	0.10	**0.90**
		**Validation**						
		G-I	**0.86**	0.14		T-IV	**0.95**	0.05
		G-III	0.10	**0.90**		T-VI	0.06	**0.94**
**Carrot cultivars**		**Calibration**						
		**Brasília**					**Francine**	
			**B-I**	**B-IV**			**F-V**	**F-VIII**
		B-I	**0.92**	0.08		F-V	**0.98**	0.02
		B-IV	0.14	**0.86**		F-VIII	0.02	**0.98**
		**Validation**						
		B-I	**0.88**	0.12		F-V	**0.97**	0.03
		B-IV	0.11	**0.89**		F-VIII	0.06	**0.94**

*G-I, T-IV, B-I, and F-V, lower-vigor seedlots; G-III, T-VI, B-IV, and F-VIII; higher-vigor seedlots. Values in bold indicate the hit rate in the quadratic discriminant analysis*.

### Photosynthetic Efficiency and Chlorophyll Content in Seedlings

**Figure 10:**
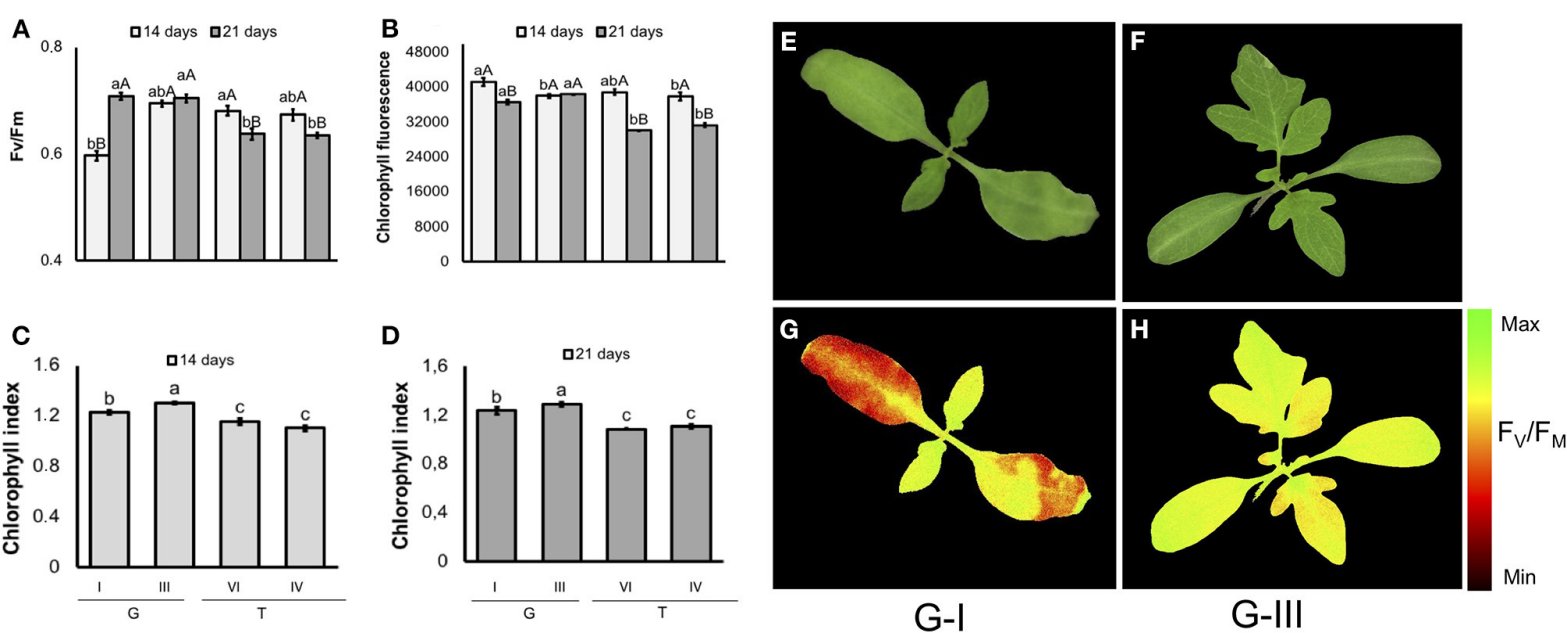
Photosynthetic activity measured by F_V_/F_M_ **(A)**, and chlorophyll fluorescence **(B)** in dark-adapted seedlings of tomato at 14 and 21 days after sowing: excitation of chlorophyll molecules was induced at 620 nm and emission at 700 nm; means (± SE) with different uppercase letters within each lot and lowercase letters within a period indicate statistically significant difference (*P* > 0.05). Chlorophyll indexes at 14 **(C)** and 21 **(D)** days were estimated from reflectance (R) values at 710 and 770 nm (R770/R710)-1; means (± SE) with a common letter are not significantly different (*P* > 0.05). Seedlings were obtained from lower-and higher-vigor seeds of Gaúcho (G) and Tyna (T) cultivars: lower-vigor = G-I and T-IV; higher-vigor = G-III and T-VI. Raw images **(E,F)** and F_V_/F_M_ images **(G,H)** were captured from 14-d-old seedlings.

In tomato (Figure 10), seeds from G-I lot generated seedlings with the lowest F_V_/F_M_ values on the 14th day of evaluation. However, G-I seedlings achieved similar values to G-III 7 days later. The F_V_/F_M_ had a cultivar-specific value in 21-d-old-seedlings: it was higher in “Gaúcho” than “Tyna,” regardless lot. Moreover, in 14-d-old seedlings from contrasting Gaúcho lots, as higher was the vigor the lower was the chlorophyll fluorescence. But, chlorophyll fluorescence reached similar values between lots as plant development progressed. Seedlings from tomato “Gaúcho” had higher chlorophyll index than “Tyna”, with different indexes between its lots (G-III had higher values than G-I).

**Figure 11:**
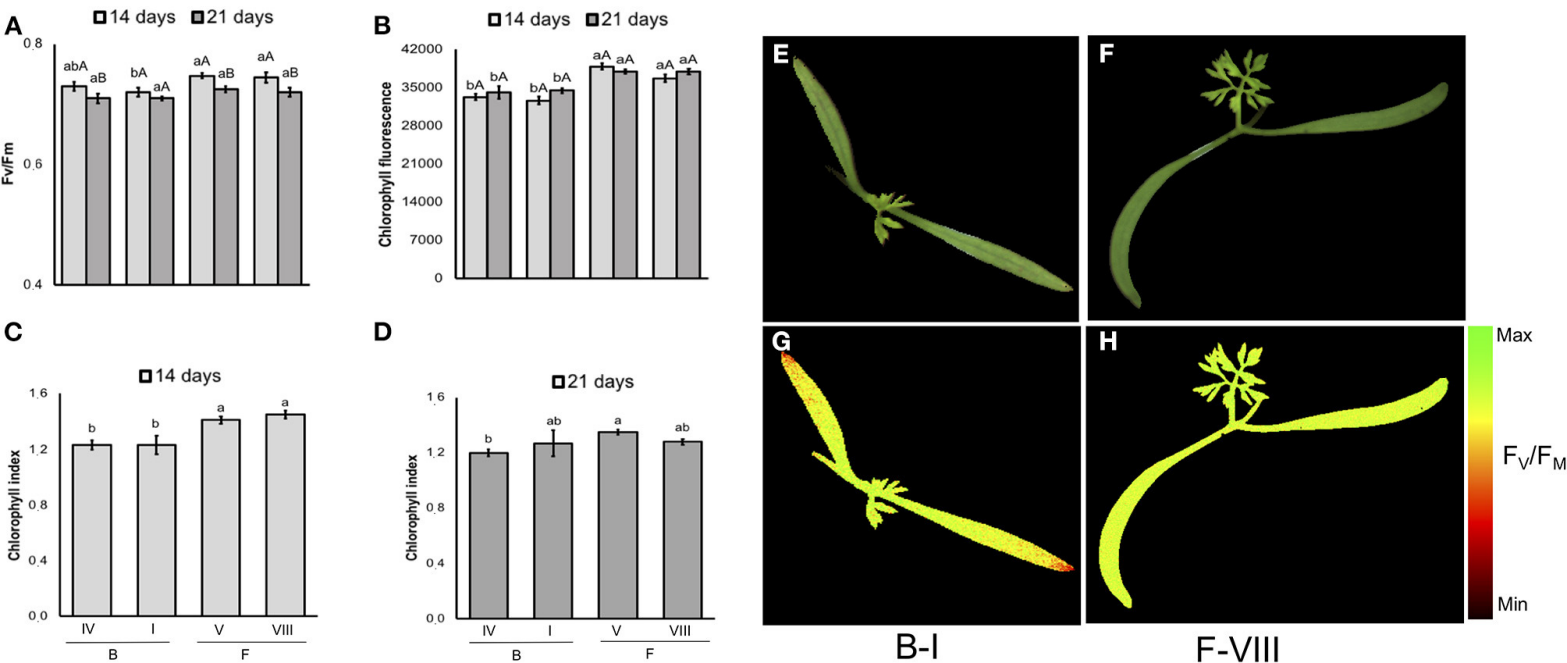
Photosynthetic activity measured by F_V_/F_M_ **(A)**, and chlorophyll fluorescence **(B)** in dark-adapted seedlings of carrot at 14 and 21 days after sowing: excitation of chlorophyll molecules was induced at 620 nm and emission at 700 nm; means (± SE) with different uppercase letters within each lot and lowercase letters within a period indicate statistically significant difference (*P* > 0.05). Chlorophyll indexes at 14 **(C)** and 21 **(D)** days were estimated from reflectance (R) at 710 and 770 nm (R770/R710)-1; means (± SE) with a common letter are not significantly different (*P* > 0.05). Seedlings were obtained from Brasília (B) and Francine (F) cultivars: B-I, B-IV, F-V and F-VIII. Raw images **(E,F)** and F_V_/F_M_ images **(G,H)** were captured from 14-d-old seedlings.

In carrot (Figure 11), although F-V lot showed the worst performance in the conventional testing method, its seeds generated seedlings with similar F_V_/F_M_ and chlorophyll index to higher-vigor seeds (e.g., F-VIII lot). In addition, a cultivar-specific value was showed for chlorophyll fluorescence and chlorophyll index: “Francine” had higher chlorophyll fluorescence signals than “Brasília” at 14 and 21 days, and higher chlorophyll index on 14-d-old-seedlings.

## Discussion

The world demand for tomato and carrot increases every year (FAOSTAT, 2020) due to their multiple utilizations (Bergougnoux, 2014), which have been further expanded by population growth, changing lifestyles, ethnic diversity, and increased income (Bergougnoux, 2014; Vidyarthi and Evans, 2019). For instance, tomato leaves are valuable sources of bioactive compounds for management of Alzheimer's disease and diabetes mellitus (Figueiredo-González et al., 2016); tomato fruits are enriched sources of relevant substances for human health (Bergougnoux, 2014) and for packaging industry (Assis et al., 2020); and tomato seeds are renewable energy sources for promising fuel substitute in diesel engines (Giannelos et al., 2005). Tomato plant is also a model organism widely used in research programs, for both applied and theoretical purposes (Bergougnoux, 2014).

Carrot is one of the most popular vegetable nowadays, being also a natural source of colorants for dyeing distinct items (such as tissues, candies, juices, and fruit preparations); polymers for fabrication of racing car steering wheel and fishing rod; oil with excellent lubricant properties for industrial applications; and antioxidants (e.g., luteolin) that can mitigate age-related inflammation and memory deficits (Jang et al., 2010; Stolarczyk and Janick, 2011). In addition, carrot is a consecrated plant model in “Life Science,” especially in studies involving cell integrity and reprogramming (Costa et al., 2009). Due to their agronomical, industrial and scientific relevancies, tomato and carrot were used to test optical imaging based on chlorophyll fluorescence and multispectral imaging technologies as non-destructive approaches for evaluation and/or prediction of the seed physiological quality.

### Chlorophyll Fluorescence and Discrimination of Crop Cultivars

Although chlorophyll is often present in developing seeds (Ooms and Destain, 2011), its content gradually decreases at the late embryogenesis stages in which chlorophyll degradation occurs in parallel with different events (such as storage of reserves, dehydration and dormancy) that are crucial for the proper seed maturation and viability (Smolikova et al., 2017). Therefore, chlorophyll can be used as a marker for seed maturity and, indirectly, quality (Kenanoglu et al., 2013). Because chlorophyll has a highly specific fluorescence [i.e., other substances which can influence seed color do not affect fluorescence signals at its specific wavelengths of excitation and emission (Jalink et al., 1998)], the chlorophyll fluorescence analysis was chosen for evaluation of tomato and carrot seeds. As shown in Figure 4, a distinct pattern of chlorophyll fluorescence could be detected in tomato and carrot cultivars when 645/700 nm excitation-emission (tomato and carrot) and 620/730 nm excitation-emission (tomato) were used. From a practical point of view, this means that chlorophyll fluorescence is a valuable marker for tomato and carrot genotypes, and it can be used as a potential tool for identification of crop cultivars.

### Chlorophyll Fluorescence and Seed Vigor Classification

The stage of seed maturity at harvest is the main factor for the success of seed production chain because fully mature seeds contain more nutrients and, consequently, higher vigor (Thompson and Kleiman, 1988; Finch-Savage and Bassel, 2015). Since tomato and carrot have continuous flowering (i.e., non-synchronous seed production), seedlots may consist of a mixture of immature and mature seeds. When seeds are harvested in their immature stage, the chlorophyll degradation is not complete and chlorophyll fluorescence intensity is modified (Kenanoglu et al., 2013). We showed that chlorophyll fluorescence-based technology clearly detected a pattern for identification of seeds with low physiological potential: the higher fluorescence, the lower vigor (Figures 4, 5). This result validates the findings of Jalink et al. (1998) who showed that the magnitude of chlorophyll fluorescence in seeds was inversely related to the germination performance and/or to the capacity for generation of normal seedlings in cabbage (*Brassica oleracea*). Seeds with non-degraded chlorophyll are more susceptible to deterioration due to the production of harmful compounds derived from chlorophyll oxidation, such as singlet oxygen (_1_O^2^) (Grulichova et al., 2018), which is toxic to cells and involved in the programmed cell death (Triantaphylidès and Havaux, 2009).

### Chemometrics-Based Multispectral Imaging for Discrimination of Crop Cultivars and Seed Vigor

We also explored multispectral imaging as a non-invasive technology for seed assessment. Principal component analysis revealed a clear characterization of carrot cultivars, and such a good separation can be explained by the strong relationship of “Francine” with the negative loadings in PC1 (Figure 7). By contrast, PCA failed to differentiate tomato cultivars (Figure 7). The last result validates the work of Shrestha et al. (2016), who reported no differentiation among five tomato cultivars using PCA on the raw visible-near infrared (Vis-NIR) spectra data.

Since the success of seed classification depends on the chemometrics method, and generally supervised approaches yield better outcomes than unsupervised ones (Shrestha et al., 2016), we applied the RF algorithm for identification of the most important bandwidths to discriminate the seed materials under study. The RF algorithm is a widely used machine learning method that generates decision trees (categorical response variable), and can combine weak classifiers to get a strong classifier, which has a high classification accuracy and strong anti-noise ability (Che et al., 2018). Accordingly, the use of the five most meaningful wavelengths assigned by the RF in a QDA-based model yielded valuable classification rates on high and low vigor seed with a correct classification from 86 to 95% in tomato and from 88 to 97% in carrot for validation set (Table 3). Interestingly, the same wavelengths selected for tomato (365, 660, 690, 570, and 590 nm, Figure 8) in this study, yielded reflectance patterns with great separation when used for discrimination of other tomato cultivars (Shrestha et al., 2015), suggesting that these bandwidths can be potentially employed as indicators of seed cultivar and/or quality in other tomato genotypes.

### Random Forest Algorithm-Selected Bandwidths in the Context of Seed Quality

It was noticed that the higher reflectance coincided with low-quality seedlots, particularly at 365 nm in tomato (Figure 8C), and at 940 or 970 nm in carrot (Figure 8D), therefore, these bandwidths can be used as proxies of the seed physiological potential in these crops. Changes in the spectral intensity may indicate that certain compounds relevant for germination and/or vigor are either in lower quantity or suffered some alteration (Xia et al., 2019). In this study, higher reflectance of low-quality seedlots was associated with immature seeds present in the lot (Figure 9). The major pigments of tomato seeds are lycopene and β-carotene (Rodriguez et al., 1975; Vági et al., 2007; Eller et al., 2010), which absorb light in the UVA/blue regions of the spectrum (Srivastava, 2002; Power et al., 2019). Thus, lower reflectance in high-quality tomato seedlots at 365 nm may be attributed to greater light-absorbing capacity of carotenoids in mature seeds. For carrot, higher reflectance of low-quality seedlots at 940 nm was perhaps influenced by lower lipid content in immature seeds because an absorbance peak at 940 nm is strongly associated with fatty tissues (Barlocco et al., 2006; Jue and Masuda, 2013). Furthermore, high absorbance at 940 and 970 nm is associated with O–H third stretching overtone that is a signature of the moisture content (Wu et al., 2008). Accordingly, Liu et al. (2016) showed variations in the moisture distribution in carrot slices as their dehydration was progressed (the higher moisture content, the lower reflectance spectra in the region from 910 to 970 nm).

Light at 365 nm can also be absorbed by phytochromes, cryptochrome and phototropins, which are photoreceptors responsible for the modulation of various physiological and developmental processes, such as germination, embryo elongation and chloroplast relocation (Jones, 2018). Phenols (Talamond et al., 2015) and lignin (Müller et al., 2013) also absorb light in the UVA region. The optimal wavelength for carotenoids estimation was also identified as 470 nm (Blackburn, 1998), so those modifications in bandwidths close to it (such as 430 nm) can be an indicative of changes in the carotenoid content. The reflectance spectra at 505, 525, 570, and 590 nm was previously selected for prediction of total anthocyanin content (Jue and Masuda, 2013; Huang et al., 2017; Sendin et al., 2018), and peaks around 675 nm represented the content of chlorophylls (Xing and De Baerdemaeker, 2005).

Although multispectral imaging can provide promising results for quality traits, this technology requires the use of calibrated systems to obtain accurate and reproducible images. For example, minor variations in the light setup, sensor sensitivity, and relative position of seeds with respect to the camera can affect the data precision (ElMasry et al., 2019). Moreover, any variation in environmental conditions (e.g., temperature and relative humidity) can cause the camera lose calibration (Mahajan et al., 2015). In the system used in this study, the light setup is first calibrated based on the type of object (e.g., tomato or carrot seed), followed by a radiometric and geometric calibration using well-defined standard targets. The purpose of radiometric calibration is to eliminate problems with uneven intensities and vignetting so that the pixel values represent actual measurement of light (Hamey, 2008). The geometric calibration is performed to ensure that geometric distortions do not affect the accuracy of the images, providing pixel correspondence for all spectra bands (Hansen, 1999; Gomez et al., 2007).

Conventional multispectral acquisition methods rely a number of different optical filters that requires longer acquisition time for changing the filters in the filter wheel or in the tunable filter device (Parmar et al., 2012). In recent years, multispectral imaging design supported with different LED illuminants has increased because this technology requires shorter acquisition time for image processing routine (ElMasry et al., 2019). Here we used an advanced multispectral imaging system with different LEDs in which the exact strobe time of each LED (maximum 123500 μs) could be optimized for each seed species and saved for all subsequent image acquisition. Individual adjustment of the light in each wavelength band results in an improved signal-to-noise ratio.

### Photosynthetic Activity and Chlorophyll Content in Seedlings Obtained From High and Low Vigor Seeds

The tracking of seedlots with contrasting vigor showed that low-quality seeds of tomato may generate seedlings with depressed photosynthetic capacity, as indicated by the low F_V_/F_M_ in G-I (Figure 10A), although in carrot the F_V_/F_M_ values were not influenced by the seed vigor within cultivar (Figure 11A). According to Baker and Rosenqvist (2004), the F_V_/F_M_ ratio estimates the maximum quantum efficiency of the photochemical activity of the PSII, and its decrease indicates a decline in the photochemical efficiency of PSII and disturbances in the photosynthetic apparatus. Because G-I presented F_V_/F_M_ values similar to the lot with higher vigor (G-III) as plant development progressed, results indicate a photosystem plasticity in G-I seedlings likely for better adaptation to the environmental conditions.

Consistently, the increased chlorophyll fluorescence in 14-day-old tomato seedlings of G-I disappeared in parallel with the progress of plant development, indicating once again that G-I seedlings are able to adjust their photosynthetic apparatus for better adaptation to the growing conditions. For chlorophyll content, a cultivar-and lot-dependent patterns were detected in tomato and carrot seedlings, in which “Gaúcho” was higher than “Tyna” tomato, and “Francine” was higher than “Brasília” carrot. These results suggest that increased chlorophyll content at seedling stage was important for better photosynthetic performance of G-III lot in “Gaúcho” tomato, and “Francine” carrot (F-V and F-VIII compared to B-I). In conclusion, both chlorophyll fluorescence and chemometrics-based multispectral imaging are powerful tools for non-invasive and reliable prediction of seed quality in both tomato and carrot. The combination of chlorophyll fluorescence and multispectral imaging technologies could contribute to discovering new biology and interpreting datasets. These approaches could also be used to meet the growing demands for food in the agricultural industry, particularly considering the high speed of computer vision systems. In addition, these approaches were able to detect seeds that yielded plants with different efficiency of energy conversion in means of photosynthesis activity. However, to increase the reliability and reproducibility of these techniques, the integration of properly calibrated systems is essential because any excessive lighting causes over saturation and incorrect measurements. Furthermore, the cost of the instrument (hardware and software) and the equipment life must be considered for building a feasible real-time optical imaging system.

## Conclusion and Future Perspectives

Optical imaging is expected to become a high priority worldwide because rapid seed quality assessment will be crucial to meet the growing demand for food in the near future. Here we bring recent advances in optical imaging based on chlorophyll fluorescence and multispectral images with great potential to improve current seed testing methods. Furthermore, these approaches can detect seedlots that will generate plants with different capacity of light conversion in photosynthesis processes.

Chlorophyll fluorescence imaging using different excitation-emission combinations is an accurate marker in the discrimination of tomato and carrot cultivars. This technique may be a useful tool in the development of new varieties in breeding programs. Multispectral imaging along with relevant multivariate chemometric analysis has high precision in distinguishing higher-vigor seeds from seeds with lower-vigor, before they are visible to the human eye. In view of the growing demand in the agricultural industry, these new approaches could potentially be used in quality assurance programs at different stages of tomato and carrot seed production, with rapid, objective, and accurate ranking of seedlots.

Our increasing knowledge about the energy-matter interaction (light and seeds with different physiological properties) may open up new possibilities for monitoring seeds in real-time with the integration of robust optical sensors. However, optical imaging is a challenging multidisciplinary science that requires expertise in computer vision, sensor development, biochemistry, physiology and statistics. Complex interaction between multiple specialties may improve the use of non-invasive diagnostic tools in modern seed technology.

## Data Availability Statement

The raw data supporting the conclusions of this article will be made available by the authors, without undue reservation.

## Author Contributions

CB generated research ideas. PG collected data and wrote the first draft. PG, MC, WH, and VB analyzed the data. PG, MC, and CB revised the draft and wrote the manuscript. MC, CB, and VA revised the manuscript for technical and scientific accuracy. All authors read and approved the final manuscript.

## Conflict of Interest

The authors declare that this research was conducted in the absence of any commercial or financial relationships that could be construed as a potential conflict of interest.
